# EC-18 prevents autoimmune arthritis by suppressing inflammatory cytokines and osteoclastogenesis

**DOI:** 10.1186/s13075-022-02941-4

**Published:** 2022-11-17

**Authors:** Jin-Sil Park, Seung Cheon Yang, Ha Yeon Jeong, Seon-Young Lee, Jun-Geol Ryu, Jeong Won Choi, Hye Yeon Kang, Sung-Min Kim, Sun-Hee Hwang, Mi-La Cho, Sung-Hwan Park

**Affiliations:** 1 The Rheumatism Research Center, Catholic Research Institute of Medical Science, The Catholic University of Korea, 222, Banpo-daero, Seocho-gu, Seoul, 06591 Republic of Korea; 2 Lab of Translational ImmunoMedicine, Catholic Research Institute of Medical Science, College of Medicine, The Catholic University of Korea, 222, Banpo-daero, Seocho-gu, Seoul, 06591 Republic of Korea; 3 Department of Biomedicine & Health Sciences, College of Medicine, The Catholic University of Korea, 222, Banpo-daero, Seocho-gu, Seoul, 06591 Republic of Korea; 4 Department of Medical Life Sciences, College of Medicine, The Catholic University of Korea, 222, Banpo-daero, Seocho-gu, Seoul, 06591 Republic of Korea; 5 Division of Rheumatology, Department of Internal Medicine, College of Medicine, Seoul St. Mary’s Hospital, The Catholic University of Korea, 222, Banpo-daero, Seocho-gu, Seoul, 06591 Republic of Korea

**Keywords:** Rheumatoid arthritis, Collagen-induced arthritis, EC-18, Inflammatory cytokines, Osteoclastogenesis

## Abstract

**Background:**

EC-18, a synthetic monoacetyldiaglyceride, exhibits protective effects against lung inflammation, allergic asthma, and abdominal sepsis. However, there have been no investigations to determine whether EC-18 has preventive potential in autoimmune diseases, especially rheumatoid arthritis (RA).

**Methods:**

To investigate the efficacy of EC-18 on the development of RA, EC-18 was administered in a collagen-induced arthritis (CIA) murine model and disease severity and the level of inflammatory cytokines in the joint were investigated. The effect of EC-18 on the inflammation-related factors was investigated by flow cytometry, ELISA, western blot, and real-time PCR in splenocytes from mice and in peripheral blood mononuclear cells from healthy and patients with RA. The effect of EC-18 on osteoclastogenesis was investigated.

**Results:**

EC-18 effectively reduced the clinical and histological severity of arthritis, similar to Janus kinase inhibitors include tofacitinib and baricitinib, in CIA. Furthermore, EC-18 exhibited a synergistic effect with methotrexate in preventing CIA. Treatment with EC-18 effectively reduced the production of inflammatory cytokines in immune cells and osteoclast differentiation in mice and patients with RA.

**Conclusion:**

These results suggest that EC-18 may be an effective strategy for RA.

## Introduction

Rheumatoid arthritis (RA) is the most prevalent chronic disease characterized by the infiltration of inflammatory cells into hyperplastic synovial tissues that results in the subsequent destruction of the adjacent articular cartilage and bone structure. This progressive immune activation and joint deterioration can lead to functional impairment, occupational disability, and decreased quality of life for patients with RA [1].

Although the pathogenesis of RA is not fully understood, numerous factors have been shown to drive the development of RA, including multiple cytokines, proteolytic enzymes, and prostanoids [1, 2]. In inflamed RA joints, proinflammatory cytokines, such as interleukin (IL)-1β, tumor necrosis factor (TNF)-α, and IL-6, produced by inflammatory cells play critical roles as inducers of prolonged local inflammation underlying the pathogenesis of RA and leading to bone destruction [2]. IL-1β and TNF-α stimulate synovial fibroblasts to secrete degradative enzymes including matrix metalloproteinases (MMPs) and cathepsins, causing articular cartilage degradation [3]. IL-17-producing T helper (Th17) cells, which are characterized by the production of their signature cytokine, IL-17A, are associated with various autoimmune diseases, including RA and multiple sclerosis [3, 4]. In RA, IL-17 activates pathogenic cells by inducing the production of proinflammatory cytokines, including IL-1β, IL-6, and TNF-α [5–7], and activated signal transducer and activator of transcription 3 (STAT3) was shown to be present in inflamed synovia in an animal model of RA [8].

Osteoclasts, which are specialized bone-resorbing multinucleated cells, are a major contributor to bone erosion in RA patients [9]. Differentiation and function of osteoclasts are promoted by macrophage colony-stimulating factor (M-CSF) and receptor activator of nuclear factor kappa-Β (NF-κB) ligand (RANKL). In addition, synoviocytes and immune cells within the RA inflamed joint produce inflammatory cytokines such as TNF-α and IL-1, which promote osteoclast differentiation and bone destruction [10, 11].

Janus kinase (JAK)/STAT pathway plays a central role in mediating signal transduction of diverse cytokines, interferons, and growth factors [12]. Recently, small molecules that inhibit JAKs are attracting attention as promising therapeutics in the treatment of RA. Tofacitinib and baricitinib are JAK inhibitors that are approved for the treatment of RA [13]. Tofacitinib is a potent pan-JAK inhibitor that specifically inhibits JAK3 and JAK1 whereas baricitinib is a selective inhibitor with higher selectivity for JAK1 and JAK2 [14, 15].

1-palmitoyl-2-linoleoyl-3-acetylglycerol (PLAG) is a monoacetyldiaglyceride that occurs naturally in a variety of seed oils and in bovine udder and milk fat [16–18]. It has also been isolated from the antlers of Sika deer (*Cervus nippon* Temminck) [19]. These antlers are one of the most important traditional oriental medicine used in East Asia more than 2000 years for the treatment of various diseases, including bone-resorbing diseases [19, 20]. Recently, EC-18 (a synthetic copy of PLAG) has been chemically synthesized from glycerol, palmitic acid, and linoleic acid and was shown to have identical efficacy to that of naturally derived monoacetyldiaglyceride [21, 22]. Previous studies showed that EC-18 exerts anti-inflammatory effects against lung inflammation, allergic asthma, and abdominal sepsis [23–25].

This study was performed to investigate the effects of EC-18 on the development of RA in a collagen-induced arthritis (CIA) mouse model. EC-18 effectively reduced the clinical and histological severity of arthritis, similar to tofacitinib and baricitinib, in CIA. Furthermore, EC-18 exhibited a synergistic effect with methotrexate (MTX) in ameliorating CIA. Treatment with EC-18 effectively reduced the production of inflammatory cytokines in immune cells and osteoclast differentiation in mice and human patients.

## Materials and methods

### Animals

Six-week-old male DBA/1J and C57BL/6 mice were purchased from Orient Bio Inc. (Seongnam, Korea). The mice were maintained under specific pathogen-free conditions at the Institute of Medical Science of the Catholic University of Korea with ad libitum access to water and were fed standard mouse chow (Ralston Purina, St. Louis, MO, USA). All experimental procedures were performed in accordance with the Laboratory Animals Welfare Act, the Guide for the Care and Use of Laboratory Animals, and the Guidelines and Policies for Rodent Experiments provided by the Department of Laboratory Animals of the Institutional Animal Care and Use Committee of the Catholic University of Korea and conformed to all National Institutes of Health (USA) guidelines (permit numbers: 2016-03-02, 2017-08-03, 2018-05-28). To minimize animal suffering, all mice were fully anesthetized under gas anesthesia using isoflurane (2–2.5%) and sacrificed by cervical dislocation.

### CIA induction and treatment with EC-18

CIA was induced as described previously [26]. To induce CIA in mice, type II collagen (CII) was dissolved overnight in 0.1 N acetic acid (4 mg/mL) with gentle rotation at 4°C. Seven-week-old male DBA/1J mice were injected intradermally at the base of the tail with 100 μg of CII emulsified in Freund’s adjuvant (Chondrex). Two weeks later, 100 μg of type II collagen dissolved and emulsified 1:1 with incomplete Freund’s adjuvant (Difco) was administered to the hind leg of mice as a booster injection. To examine the preventive effects of EC-18 on the development of arthritis, mice were administered via oral gavage with the vehicle only (2.5 % DMSO in PBS), baricitinib (3 mg/kg, 0.4 % DMSO in PBS), tofacitinib (15 mg/kg, 1.2 % DMSO in PBS), or EC-18 (250 mg/kg, 2.5 % DMSO in PBS), which was developed as an oral treatment, once daily from day 21 after the first immunization. To evaluate the synergistic effects of EC-18 with MTX, mice were administered via oral gavage with the vehicle only (2.5 % DMSO in PBS), MTX (1.5 mg/kg, 0.1 % DMSO in PBS), EC-18 (125 mg/kg, 2.5 % DMSO in PBS), or MTX combined with EC-18 once daily from day 21 after the first immunization. The mice were scored using the arthritis index, which indicates the onset and severity of joint inflammation, twice weekly for up to 8–10 weeks after the first immunization.

### Clinical assessment of arthritis

To assess the severity of joint inflammation, the arthritis index was scored twice weekly from the onset of arthritis for up to 8–10 weeks after the primary immunization. The severity of arthritis was assessed on a scale of 0–4 on 4 paws per mouse (maximum score of 16) according to the following criteria, as described previously [27]: 0 = no edema or swelling, 1 = slight edema and erythema limited to the foot or ankle, 2 = slight edema and erythema from the ankle to the tarsal bone, 3 = moderate edema and erythema from the ankle to the tarsal bone, and 4 = edema and erythema from the ankle to the entire leg. Incidence was calculated as 25% of the presence of arthritis symptoms in one foot. The arthritic score and incidence for each mouse were expressed as the sum of the scores of four limbs.

### Antibodies

The following antibodies were used for immunohistochemistry: rabbit polyclonal anti-IL-6 (1:50, ab7737), rabbit polyclonal anti-TNF-α (1:150, ab6671), rabbit polyclonal anti-IL-17 (1:600, ab79056), rabbit monoclonal anti-STAT3 (phosphor Tyr705) (1:100, ab76315), rabbit monoclonal anti-tartrate-resistant acid phosphatase (TRAP) (1: 100, ab191406), and rabbit IgG, monoclonal [EPR25A] isotype control (1:100, ab172730) were from abcam; rabbit polyclonal anti-IL-1β Ab (1:400, NB600-633) was from Novus Biologicals. The following antibodies were used for western blot: mouse monoclonal anti-β-actin Ab (1:1000, sc-47778) were from Santa Cruz Biotechnology; rabbit monoclonal anti-STAT3 (phosphor Y705) (1:2000, #9145), rabbit polyclonal anti-STAT3 (phosphor S727) (1:1000, #9134), and mouse monoclonal STAT3 (124H6) Ab (1:1000, #9139) were from Cell signaling; rabbit monoclonal anti-GAPDH [EPR16891] (1:2000, ab181602) was from abcam. The following antibodies were used for flow cytometry for mouse: rat monoclonal PerCP-Cyanine5.5 anti-CD4 (0.25 μg/test, #45-0042-82) and rat monoclonal FITC anti-IL-17A Ab (0.25 μg/test, #11-7177-81) were from Thermo Fisher Scientific; rat PE/Cyanine5 IgG2a κ isotype control (#400510) and rat FITC IgG2a, κ isotype control Ab (#400505) were from BioLegend. The following antibodies were used for flow cytometry for human: mouse monoclonal PE/Cyanine7 anti-human CD4 (5 μl per test, #300512) and rat monoclonal FITC IgG2a, κ isotype control Ab (#400505) were from BioLegend; mouse monoclonal PE anti-human IL-17 (0.25 μg/test, #12-7179-42), mouse monoclonal APC anti-CD25 (0.125 μg/test, #17-0259-42), rat monoclonal FITC anti-Foxp3 (0.5 μg/test, #11-4776-42), mouse PE-Cyanine7 IgG1, κ isotype control (# 25-4714-42), mouse PE IgG1, κ isotype control (#12-4714-42), and mouse APC IgG1, and κ isotype control Ab (#17-4714-42) were from Thermo Fisher Scientific.

### Histopathology

Mouse joint tissues were fixed in 10% neutral-buffered formalin, decalcified in decalcifying agent (National Diagnostics, Atlanta, GA, USA), embedded in paraffin, and cut into sections 5 μm thick. The sections of ankle/tarsal bones were stained with hematoxylin and eosin (H&E) and safranin O and scored as described previously [28]. Inflammation was scored using the following criteria: 0, normal; 1, minimal infiltration of inflammatory cells and/or mild edema; 2, mild infiltration; 3, moderate infiltration; 4, marked infiltration; and 5, severe infiltration of the synovium by inflammatory cells. Bone erosion was scored using the following criteria: 0, normal; 1, clearly invisible bone resorption in trabecular and cortical bone; 2, clearly invisible bone resorption in slightly increased trabecular and cortical bone; 3, clearly visible bone resorption in the trabecular and cortical bones. Cortex thickness decreases and trabecular loss occurs; 4, no distortion on the cortical bone surface and the thickness of trabecular and cortical bone is reduced; 5, decreased cortical bone surface and thickness of trabecular and cortical bone. Cartilage damage was evaluated by staining with Safranin O and toluidine blue, and the extent of damage was scored using the following criteria: 0, normal; 1, minimal loss of cartilage without apparent chondrocyte loss or collagen disruption; 2, chondrocyte loss and/or collagen disruption and mild loss of cartilage (superficial); 3, moderate loss of chondrocyte loss and/or collagen disruption and cartilage (depth to middle zone); 4, marked loss of chondrocyte loss in various areas and/or collagen disruption and cartilage (depth to deep zone); and 5, severe chondrocyte loss and/or collagen disruption and (depth to tidemark) severe loss.

### Immunohistochemistry

Sections were treated with 3% (v/v) H_2_O_2_ in methanol to block endogenous peroxidase activity. Immunohistochemistry was performed using the Envision Detection™ kit (DAKO Agilent Technologies Inc., Santa Clara, CA, USA). Tissue sections were incubated with primary antibodies against IL-6, TNF-α, IL-1β, IL-17, phosphorylated (*p*) STAT3 (Tyr705), and TRAP for 2 h at room temperature. Sections were then incubated with a biotinylated secondary Ab and streptavidin–peroxidase complex for 30 min. The final colored products were developed using chromogen diaminobenzidine, and the sections were examined under a photomicroscope (Olympus, Tokyo, Japan). Two independent, blinded observers assessed the number of positive cells per section at high-power field (magnifications ×400). To analyze osteoclast parameters, osteoclast surface normalized to the bone surface were assessed by bone histomorphometric analyses (ImageJ software).

### Isolation of splenocytes and cell stimulation

Splenocytes and splenic CD4^+^ cells from normal C57BL/6 or CIA mice were isolated as described previously [29]. Mouse spleens were ground using sterilized glass slides with frosted ends and red blood cells were lysed in hypotonic ACK buffer (0.15 mM NH_4_Cl, 1 mM KCO_3_, and 0.1 mM EDTA, pH 7.4). The remaining splenocytes were filtered through a 40-μm cell strainer (Falcon, Durham, NC) and maintained in RPMI 1640 medium containing 5% fetal bovine serum (Thermo Fisher Scientific, MA, USA). To purify splenic CD4+ T cells, the splenocytes were incubated with CD4-coated magnetic beads and isolated on MACS separation columns (Miltenyi Biotec). Splenocytes were pretreated with EC-18 for 2 h and then stimulated with 100 ng/ml lipopolysaccharide (LPS) (Sigma-Aldrich #L4391) for 3 days. Th0 cells were stimulated only with 0.5 μg/ml anti-CD3 (BD Pharmingen #553057) and 1 μg/ml anti-CD28 (BD Pharmingen #553294) antibodies without the addition of cytokines. Th17 differentiation was induced via treatment with anti-CD3, anti-CD28, 20 ng/ml IL-6 (R&D systems #406-ML), 2 ng/ml transforming growth factor (TGF)-β (PeproTech #100-21C), 5 μg/ml anti-IFN-γ Ab (R&D systems #MAB485), and 5 μg/ml anti-IL-4 Ab (R&D systems #MAB404). For regulatory T (Treg) polarization, anti-CD3, anti-CD28, anti-IFN-γ anti-IL-4 antibodies, and TGF-β (5 ng/ml) were added to the culture. Splenic CD4^+^ cells were pretreated with EC-18 for 2 h and then stimulated under Th0, Th17, or Treg conditions for 3 days. To determine the levels of *p*- STAT3 by western blot, CD4^+^ cells were stimulated with IL-6 for 30 min or anti-CD3 and anti-CD28 Ab for 12 h in the presence of EC-18.

### Isolation of human peripheral blood mononuclear cells and cell stimulation

Human blood was obtained from patients with RA (Table 1). The diagnosis of RA was confirmed according to the revised criteria of the American College of Rheumatology [30]. Normal healthy volunteers were included as controls. All patients provided informed consent in accordance with the guidelines of the Declaration of Helsinki. Approval by the ethics committee of Seoul St. Mary’s Hospital (Seoul, Korea) was obtained for all procedures (permit numbers: KC17MNSI0405, KC17TNSI0704). Peripheral blood mononuclear cells (PBMCs) were separated from the buffy coat using Ficoll–Hypaque (Pharmacia Biotech, Piscataway, NJ, USA). The cells were pretreated with EC-18 for 2 h and then stimulated with anti-CD3 Ab (0.5 μg/ml) for 12 h, 2 days, or 3 days.

**Table 1:** Clinical characteristics of patients with rheumatoid arthritis

Patient number	1	2	3	4
Age	47	54	64	41
Sex	M	F	F	F
Body mass index	25.0	25.5	22.0	21.7
Disease duration	72 months	176 months	140 months	281 months
RF	175.9	199.6	108.3	72.3
Anti-CCP titer	340	100	80	7.6
DAS28-CRP	2.48	2.3	2.45	3.32
ESR	25	3	4	19
CRP	0.20	0.29	0.38	0.47
Medication	MTX + tocilizumab + prednisolone 5mg	MTX	MTX	MTX + tacrolimus +prednisolone 5mg
Comorbid disease	Hyperlipidemia	Osteoporosis	Diabetes mellitus, osteoporosis, hyperlipidemia	Osteoporosis

*M* male, *F* female, *mo* month, *RF* rheumatoid factor (normal < 14 IU/ml), *anti-CCP* anti-cyclic citrullinated peptide antibody (normal < 5 units/ml), *DAS28* disease activity score 28, *CRP* c-reactive protein (mg/dL), *ESR* erythrocyte sedimentation rate (mm/h), *MTX* methotrexate

### Mouse in vitro osteoclastogenesis

Bone marrow-derived monocytes/macrophages (BMMs) were isolated from the tibias and femurs of the mice by flushing the bone marrow cavity with α-minimum essential medium (MEM; Invitrogen, Carlsbad, CA, USA). The cells were incubated with α-MEM containing 10% fetal bovine serum for 12 h to separate the floating and adherent cells. Floating cells (1 × 10^5^ cells/500 μl) were cultured in the presence of M-CSF (10 ng/ml) for 3 days to form osteoclast precursor cells (preosteoclasts). Three days later, the nonadherent cells were washed out, and preosteoclasts were stimulated with EC-18 in the presence of 10 ng/ml macrophage colony-stimulating factor (M-CSF, PeproTech #300-25) and 50 ng/ml RANKL (PeproTech #310-01) for 4 days to generate osteoclasts. On day 2, the medium was replaced with fresh medium containing M-CSF, RANKL, and EC-18. TRAP staining (Cosmo Bio, #PMC-AK04F) was performed according to the manufacturer’s instructions. Mouse BMMs were prepared using the method described above and were cultured in 48-well OAAS plates (Osteogenic Core Technologies, Choongnam, Korea). Erosive areas were identified using the Tomoro analySIS TS Lite program (Olympus, Münster, Germany).

### Human in vitro osteoclastogenesis

PBMCs (5 × 10^5^ cells/500 μl) were incubated at 37°C for 2 h to separate nonadherent and adherent cells, and the adherent cells were then cultured with M-CSF (100 ng/ml) for 3 days. After 3 days, these preosteoclast cells were cultured further in the presence of M-CSF (25 ng/ml), RANKL (30 ng/ml), and EC-18 for 9 days to generate osteoclasts. On day 3, the medium was replaced with fresh medium containing M-CSF, RANKL, and EC-18. TRAP staining (Cosmo Bio) was performed according to the manufacturer’s instructions.

### Analysis of gene expression using real-time polymerase chain reaction (PCR)

Total RNA was extracted using TRI Reagent (Molecular Research Center), and cDNA was synthesized with Dyne first-strand cDNA synthesis kit (Dyne Bio) according to the manufacturer’s protocol. Polymerase chain reaction amplification and analysis were performed using a LightCycler 2.0 instrument (Roche Diagnostics, Indianapolis, IN, USA) and the accompanying software (version 4.0). All reactions were performed using a SensiFAST™ SYBR® Hi-ROX kit (Meridian Bioscience, Cincinnati, OH, USA), according to the manufacturer’s instructions. The following primers were used for mouse sequences: TRAP, 5′-TCCTGGCTCAAAAAGCAGTT-3′ (sense), 5′-ACATAGCCCACACCGTTCTC-3′ (antisense); carbonic anhydrase II, 5′-TGGTTCACTGGAACACCAAA-3′ (sense), 5′-AGCAAGGGTCGAAGTTAGCA-3′ (antisense); calcitonin receptor, 5′-CGGACTTTGACACAGCAGAA-3′ (sense), 5′-AGCAGCAATCGACAAGGAGT-3′ (antisense); and β-actin, 5′-GTACGACCAGAGGCATACAGG-3′ (sense), 5′-GATGACGATATCGCTGCGCTG-3′ (antisense). The following primers were used for human samples: RORc, 5′-AGTCGGAAGGCAAGATCAGA-3′ (sense), 5′-CAAGAGAGGTTCTGGGCAAG-3′ (antisense); Foxp3, 5′-CACTGCCCCTAGTCATGGT-3′ (sense), 5′-GGAGGAGTGCCTGTAAGTGG-3′ (antisense); and β-actin, 5′-GGACTTCGAGCAAGAGATGG-3′ (sense), 5′-TGTGTTGGGGTACAGGTCTTTG-3′ (antisense). The levels of mRNA expression were normalized relative to that of β-actin mRNA.

### Enzyme-linked immunosorbent assay (ELISA)

The levels of IL-6, IL-1β, TNF-α, IL-17, and IL-10 for mouse (DY406, DY401, DY410, DY421, DY417) or human (DY206, DY201, DY210, DY317, DY217B) were performed using DuoSet ELISA kits (R&D Systems). HRP–avidin (R&D Systems) was used for color development. The absorbance of each sample was measured at 405 nm (A_405_) using an ELISA microplate reader (Molecular Devices, Sunnyvale, CA, USA).

### Measurement of IgG2a and type II collagen-specific IgG2a

Blood was taken from the orbital sinus of mice, and the sera were stored at -20°C until use. The concentrations of IgG2a and anti-CII-specific IgG2a antibodies were measured using a mouse IgG2a ELISA quantification kit (Bethyl Lab Co., Montgomery, TX, USA). The absorbance at 405 nm was measured with an ELISA microplate reader (Molecular Devices).

### Flow cytometry

For surface marker staining, single-cell suspensions were washed with FACS buffer and incubated with fluorochrome labeled-antibodies for 30 min at 4°C. For intracellular staining, single-cell suspensions were cultured with 25 ng/ml PMA (Sigma-Aldrich, p8139) and 250 ng/ml ionomycin (Sigma-Aldrich, I0634) with the addition of GolgiStop (BD Biosciences, #554715) for 4 h. After surface staining, cells were fixed and permeabilized with Cytofix/Cytoperm according to the manufacturer’s instructions (BD Biosciences, #554715). For intracellular Foxp3 staining, a Foxp3/Transcription Factor Staining Buffer kit was used (Invitrogen, #00-5523-00) after surface staining. After washing with Perm/Wash buffer, antibodies for intracellular staining were added for 30 min at 4°C. To identify murine Th17 cells, the cells were intracellularly stained with anti-IL-17-FITC Ab, followed by anti-CD4–PerCPCy5.5 Ab. To identify human Th17 cells, the cells were intracellularly stained with anti-IL-17-PE Ab, followed by anti-CD4-PE/Cyanine7 Ab. To identify human Treg cells, the cells were intracellularly stained with anti-Foxp3-FITC Ab, followed by anti-CD4-PerCP and anti-CD25-APC Ab. Stained cells were analyzed on a FACSCalibur (BD Biosciences) or CytoFLEX (Beckman Coulter). Events were collected and analyzed with FlowJo software (Tree Star).

### Western blot analysis

Cells were lysed in Halt protein lysis buffer containing Halt phosphatase inhibitor (Thermo Scientific Pierce, Waltham, MA, USA), and protein concentrations were determined using the Bradford protein assay (Bio-Rad, Hercules, CA, USA). Proteins were separated using sodium dodecyl sulfate-polyacrylamide gel electrophoresis, transferred onto Hybond ECL membranes (GE Healthcare, Waukesha, WI, USA), and incubated with antibodies against *p*-STAT3 (Tyr705, Ser727), STAT3, and β-actin or GAPDH for 10 min using the SNAP i.d. Protein Detection System (Millipore, Billerica, MA, USA). After washing, HRP-conjugated secondary Ab was added and incubated for 10 min. Ab binding was detected using an enhanced chemiluminescence detection kit (Pierce, Rockford, IL, USA) and Hyperfilm (Agfa, Mortsel, Belgium). Images were scanned on an Epson Perfection V700 photo scanner and quantification of intensity was performed using an ImageJ software. The levels of interest protein were normalized relative to that of β-actin or GAPDH as a loading control, and relative level of interest protein at nil condition was considered as 1.

### Statistical analysis

Statistical analyses were performed using GraphPad Prism, version 8 for Windows (GraphPad Software). *P* values were calculated using the two-tailed paired *t*-test and two-way analysis of variance (grouped). *P* < 0.05 was considered statistically significant. *P* values are presented within each figure or figure legend.

## Results

### EC-18 prevents the severity of experimental arthritis

**Fig. 1:**
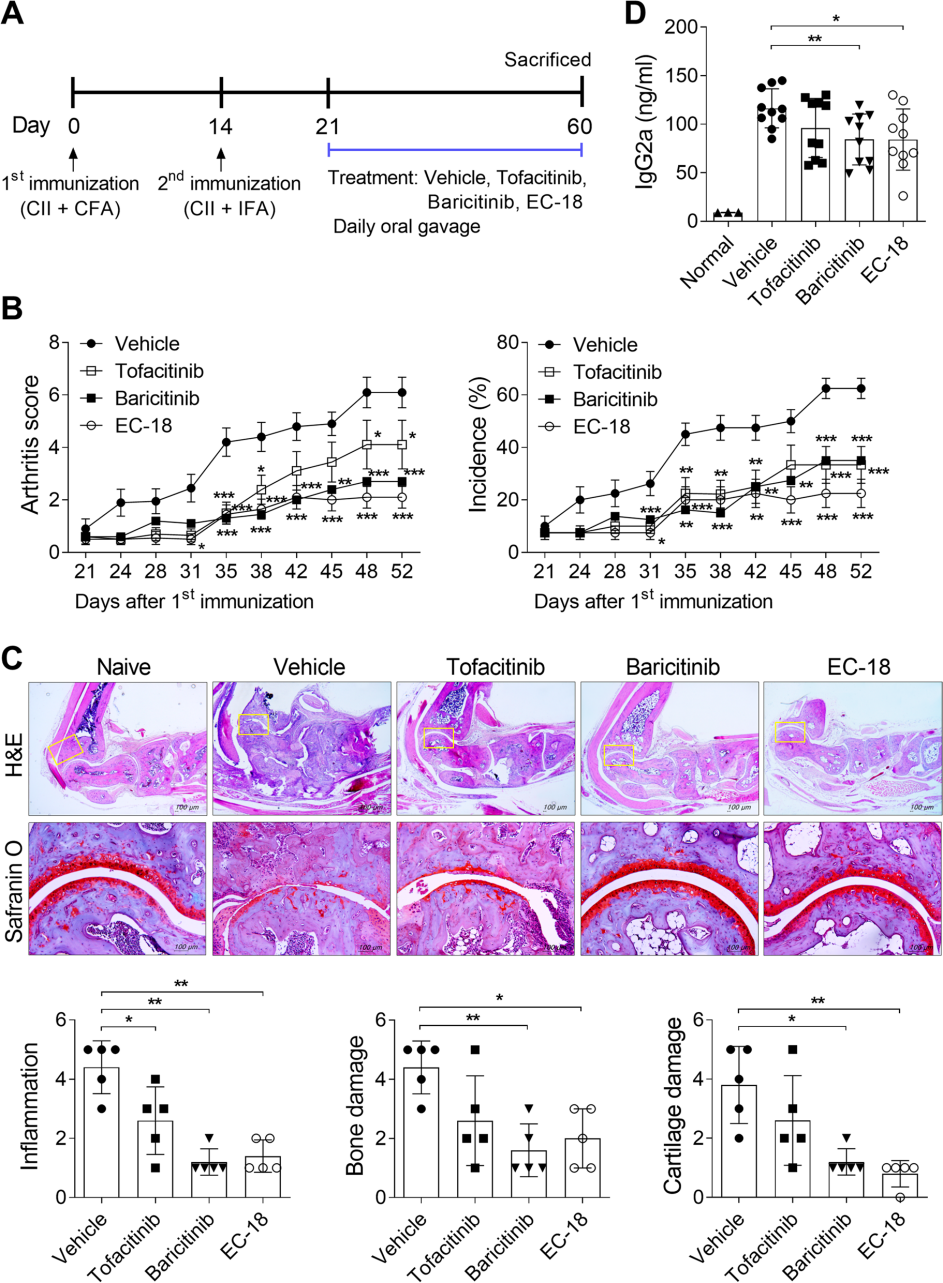
Treatment with EC-18 ameliorated the severity of experimental arthritis. The vehicle only, tofacitinib (15 mg/kg), baricitinib (3 mg/kg), or EC-18 (250 mg/kg) was orally administered to mice once daily from day 21 after the first immunization (*n* = 10/group). **A** A graphic scheme of CIA induction and drug administration. **B** Arthritis development was assessed based on the arthritis score (left) and incidence (right) (*n* = 10/group). The severity of arthritis was assessed on a scale of 0–4 according to the criteria and incidence was calculated as 25% for one foot with arthritis symptoms. *P < 0.05, **P < 0.01, ***P < 0.001 *vs*. vehicle-treated control group by two-way ANOVA (Bonferroni posttests). **C** At 60 days after the first immunization with type II collagen (CII), sections of ankle joint tissue of subjects with the average arthritis score of each group (*n* = 5/group) were stained with hematoxylin and eosin (H&E) (original magnification ×40) and safranin O (original magnification ×200). Joint tissue from normal mice was used as a control. Representative histological features are shown. Two independent, blinded observers assessed the cartilage damage, inflammation, and bone damage was assessed on a scale of 0–4 according to the criteria. **D** At 60 days after the first immunization, serum level of IgG2a was measured via enzyme-linked immunosorbent assay (ELISA). *P < 0.05, **P < 0.01, ***P < 0.001 *vs*. vehicle-treated control group

**Fig. 2:**
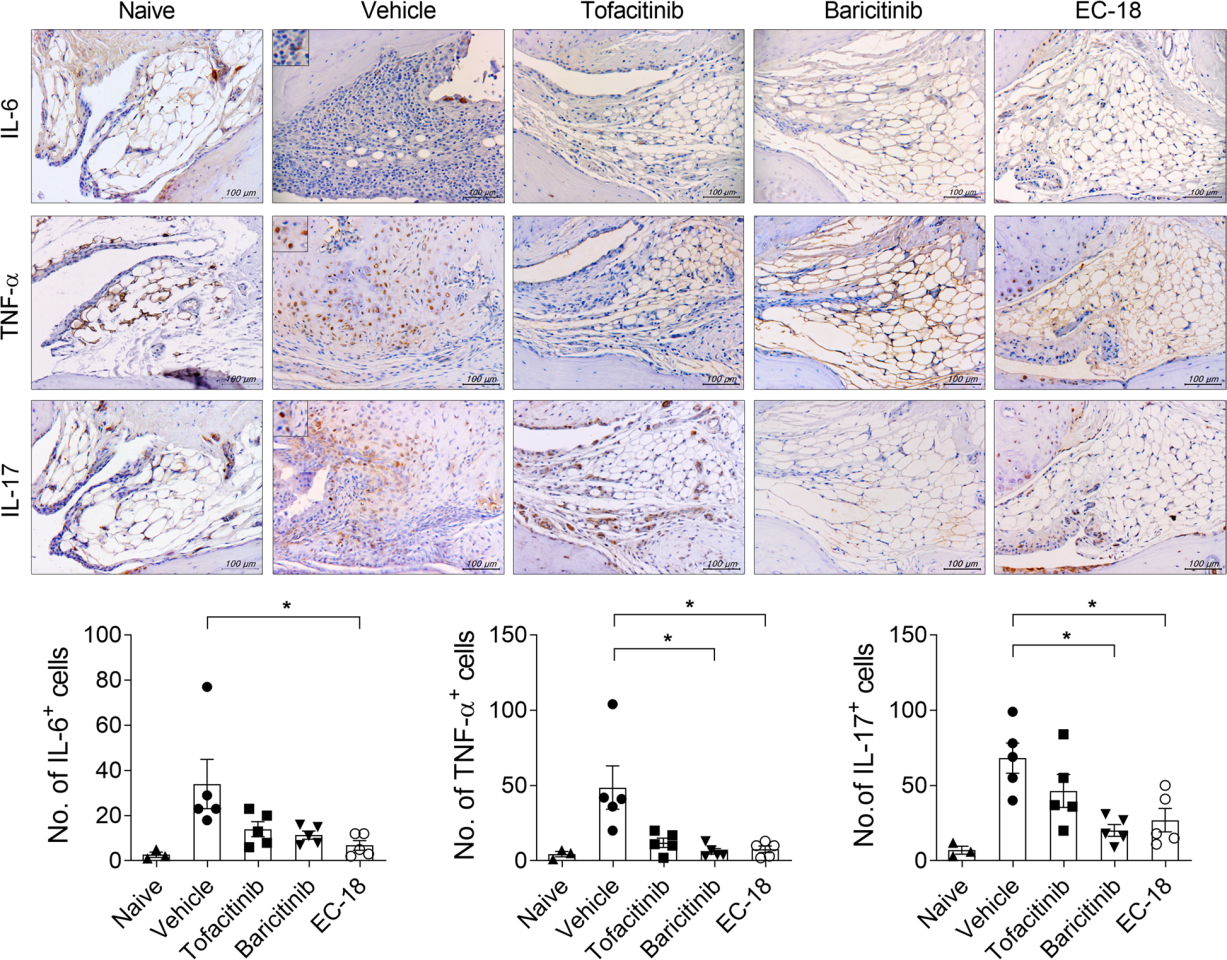
Treatment with EC-18 suppressed the levels of inflammatory cytokines in vivo. The vehicle only, tofacitinib (15 mg/kg), baricitinib (3 mg/kg), or EC-18 (250 mg/kg) was orally administered to mice once daily from day 21 after the first immunization. At 60 days after the first immunization with CII, the mice were sacrificed and examined to assess changes in the number of cytokine-positive cells in joint synovial membrane. Sections of joint tissues of subjects with the average arthritis score of each group (*n* = 5/group) were stained with antibodies against interleukin (IL)-6, tumor necrosis factor (TNF)-α, and IL-17 (original magnification ×400). Joint tissue from normal mice was used as a control (*n* = 3). The graphs present the number of Ab-positive cells for each cytokine. *P < 0.05 *vs*. vehicle-treated control group

To examine whether EC-18 could prevent the development of arthritis in vivo, EC-18 was orally administered to CIA once daily from day 21 after CII immunization (Fig. 1A). Tofacitinib and baricitinib were used as positive controls for EC-18. EC-18 effectively reduced both the arthritis score and disease incidence of arthritis, similar to tofacitinib and baricitinib, in CIA (Fig. 1B). Histological examination of joints stained with H&E and safranin O showed that the paws and ankles of EC-18-treated mice exhibited less severe inflammation, bone damage, and cartilage damage compared with the vehicle-treated control group (Fig. 1C). The amount of IgG2a was lower in the serum of mice treated with EC-18 or baricitinib compared to that in vehicle-treated controls (Fig. 1D). The joints of experimental arthritic mice treated with baricitinib also exhibited control of joint damage and inflammation, similar to the EC-18 group. To assess the anti-arthritic effects of EC-18, joint sections were immunostained for inflammatory cytokines, including IL-6, TNF-α, and IL-17. EC-18 treatment reduced the levels of these inflammatory cytokines in joint synovial membrane from EC-18-treated mice compared with those in vehicle-treated controls (Fig. 2).

### Combined treatment with EC-18 and MTX effectively prevented experimental arthritis

**Fig. 3:**
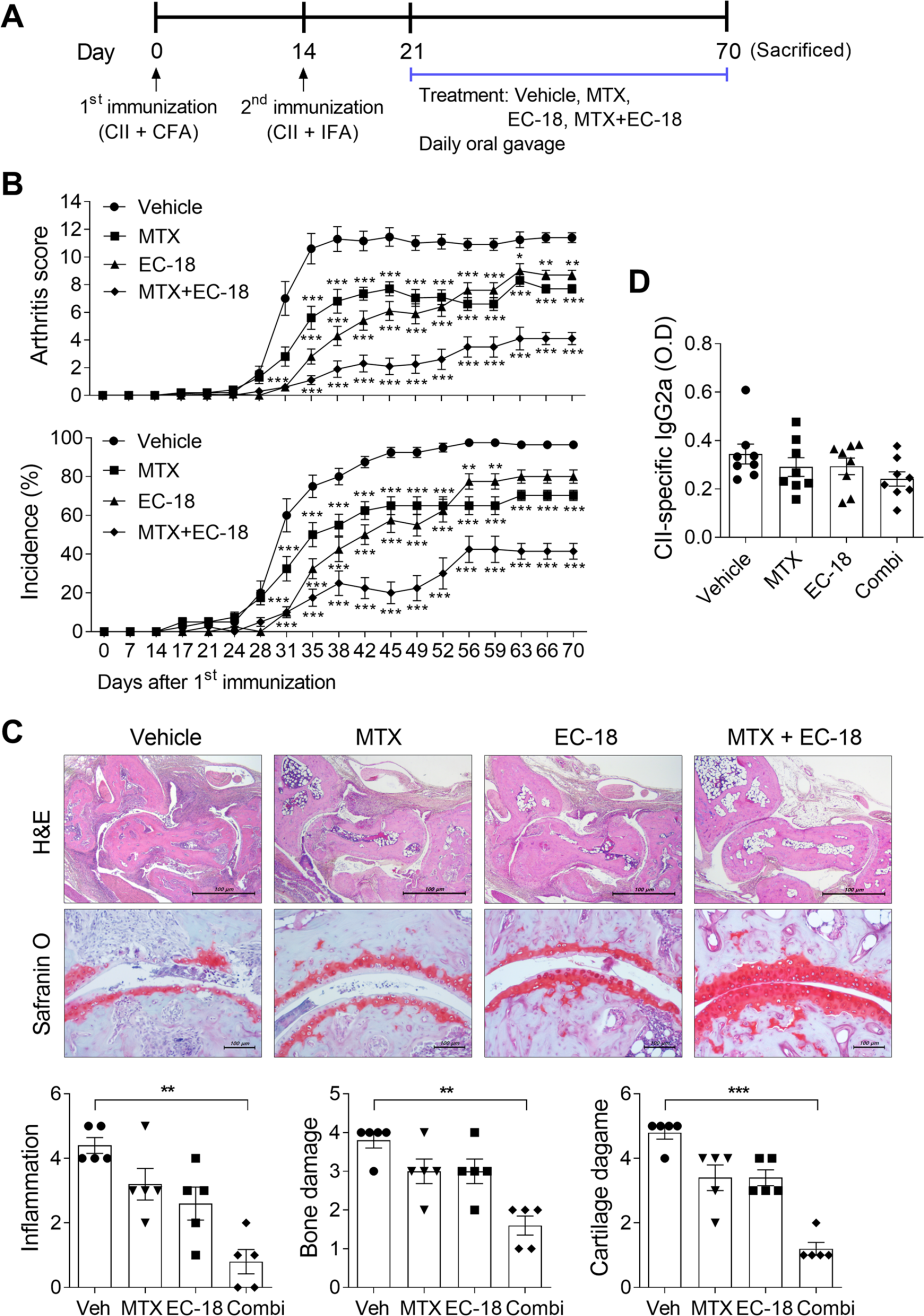
EC-18 combined with methotrexate attenuated the severity of collagen-induced arthritis. The vehicle only or EC-18 (125 mg/kg) was administered to mice once daily from day 21 after the first immunization, and methotrexate (1.5 mg/kg) was administered three times a week by oral feeding (*n* = 10/group). **A** A graphic scheme of CIA induction and drug administration. **B** Arthritis development was assessed based on the arthritis score (upper) and incidence (lower). *P < 0.05, **P < 0.01, ***P < 0.001 *vs*. vehicle-treated control group by two-way ANOVA (Bonferroni posttests). **C** At 70 days after the first immunization with CII, sections of ankle joint tissues of subjects with the average arthritis score of each group (*n* = 5/group) were stained with H&E (original magnification ×40) and safranin O (original magnification ×200). Representative histological features are shown. Two independent, blinded observers assessed the cartilage damage, inflammation, and bone damage was assessed on a scale of 0–4 according to the criteria. **D** At 70 days after the first immunization, serum level of CII-specific IgG2a were measured via enzyme-linked immunosorbent assay (ELISA) (*n* = 8/group). *P < 0.05, **P < 0.01, ***P < 0.001 *vs*. vehicle-treated control group

**Fig. 4:**
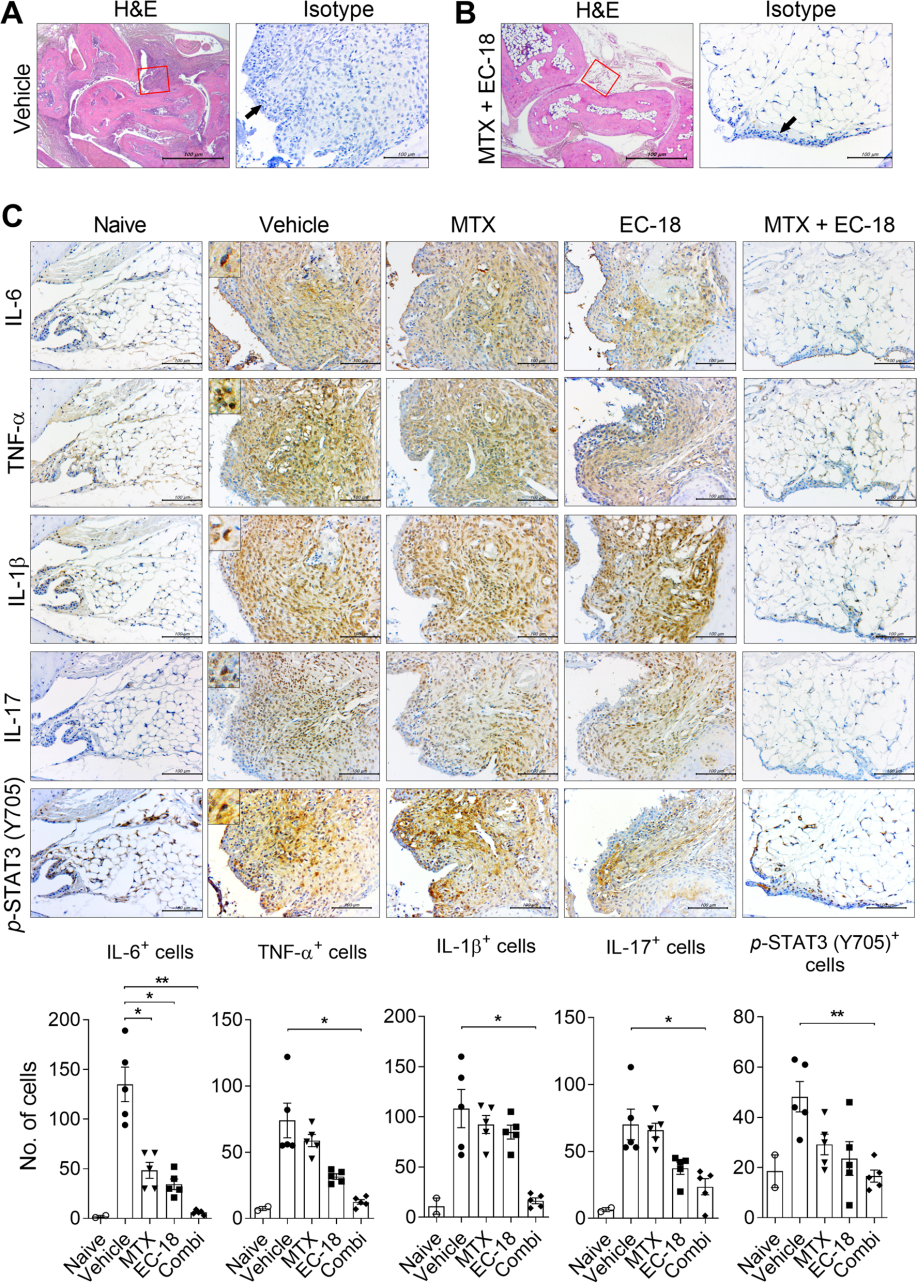
EC-18 combined with methotrexate reduced the levels of inflammatory cytokines in vivo. The vehicle only or EC-18 (125 mg/kg) was administered to mice once daily from day 21 after the first immunization, and methotrexate (1.5 mg/kg) was administered three times a week by oral feeding. At 70 days after the first immunization with CII, the mice were sacrificed and examined to assess changes in the levels of inflammatory cytokines in joint tissues of subjects with the average arthritis score of each group (*n* = 5/group). **A, B** H&E and isotype staining for immunohistochemistry of joint sections from vehicle-treated (**A**) or EC-18 plus MTX-treated (**B**) CIA mice. Rabbit monoclonal Ab for immunohistochemistry was used as isotype control. Immunohistochemistry was performed on the areas delineated by a box in H&E images. Arrows indicate synovium. **C** Sections of joint tissues were stained with antibodies against IL-6, TNF-α, IL-1β, IL-17, and *p*-STAT3 (Tyr705) (original magnification ×400). Joint tissue from normal mice was used as a control (*n* = 2). The graphs present the number of Ab-positive cells for each cytokine. *P < 0.05, **P < 0.01 *vs*. vehicle-treated control group

MTX is a first-line disease-modifying antirheumatic drug (DMARD) commonly used in RA [31]. Conventional treatment of RA is usually based on low doses of MTX [32]. To examine whether EC-18 has synergistic effects with MTX in the prevention of CIA, we compared the modulating effects of EC-18 alone and EC-18 combined with MTX on the severity of arthritis in a mouse model (Fig. 3A). To investigate the additive effect when treated with MTX, 125 mg/kg EC-18, which is half of the dose used in Fig. 1, was administered. As shown in Fig. 3B, EC-18 effectively controlled the development of experimental arthritis, and the effect was maintained until 10 weeks after the first immunization, which was similar to the therapeutic efficacy of MTX. Notably, combined treatment with EC-18 and MTX suppressed the experimental arthritis score and incidence to a much greater extent than either of these drugs alone (Fig. 3B). Histological examination of joints revealed that combination therapy with EC-18 and MTX resulted in decreased infiltration of inflammatory cells and less severe cartilage loss in CIA than treatment with the vehicle only (Fig. 3C). The level of CII-specific IgG2a was lower in the serum of mice treated with EC-18 plus MTX compared to that in vehicle-treated controls, but there was no statistical significance (Fig. 3D). Furthermore, the joints of EC-18 plus MTX-treated CIA mice had fewer IL-6-, TNF-α-, IL-1β-, IL-17-, and STAT3-positive cells compared to those of vehicle-treated controls (Fig. 4C). Figure 4A and B show the location of synovium (red line box) and isotype control staining images for IHC in the joint of vehicle or MTX and EC-18 combined treatment group, respectively.

### EC-18 reduces the production of IL-17 in mice in vitro

**Fig. 5:**
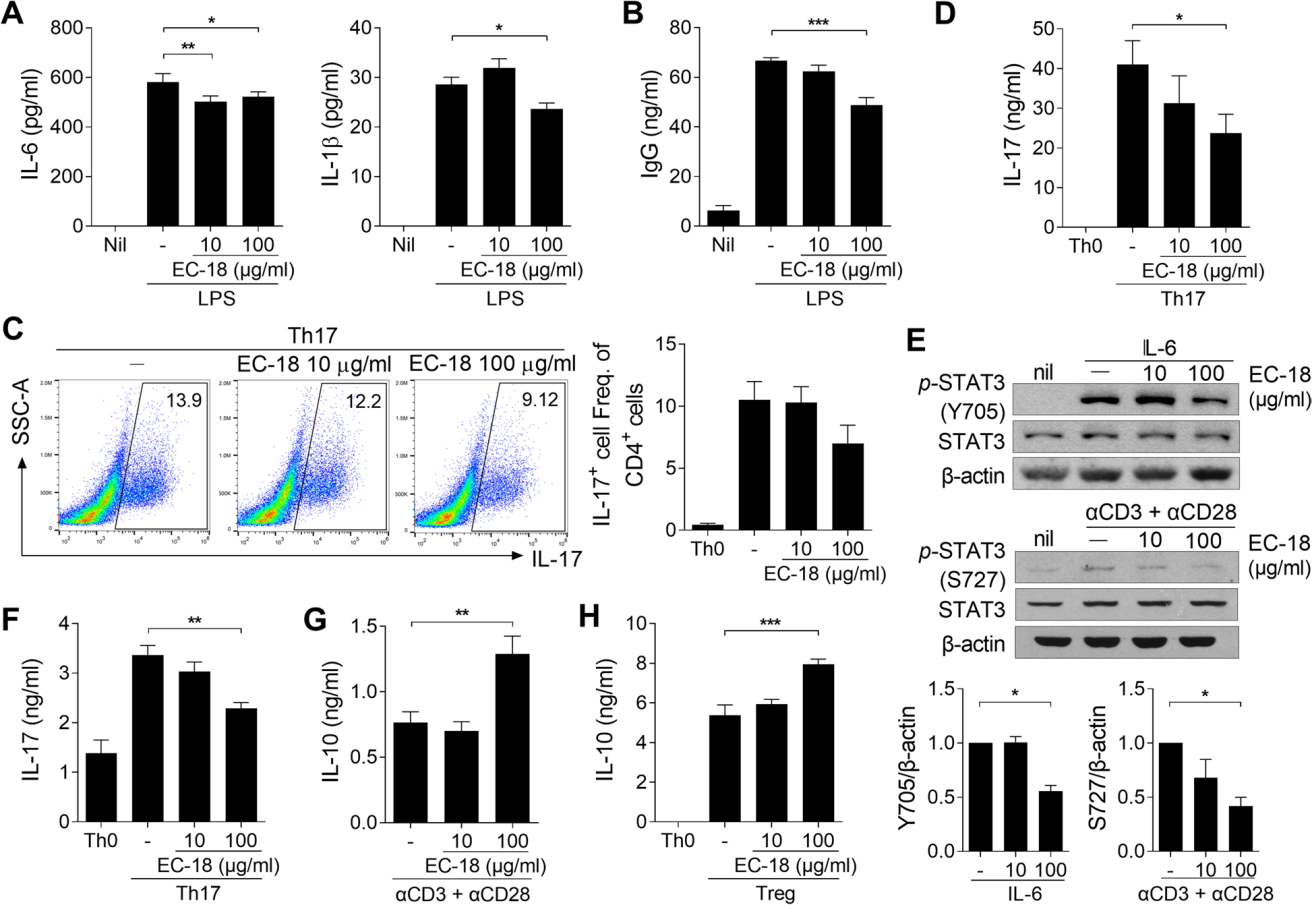
Treatment with EC-18 decreased the production of inflammatory mediators in vitro. **A, B** Splenocytes (1 × 10^6^ cells/ml) from C57BL/6 mice were stimulated with EC-18 (10 or 100 μg/ml) or vehicle for 2 h and then untreated (Nil) or treated with lipopolysaccharide (LPS; 100 ng/ml) for 3 days. Levels of IL-6, IL-1β (**A**) and IgG (**B**) in the culture supernatant were determined using ELISA. **C, D** Splenic CD4^+^ cells (5 × 10^5^ cells/500 μl) were stimulated with EC-18 (10 or 100 μg/ml) or vehicle for 2 h and then cultured under IL-17-producing T helper (Th17) cell-activating conditions with anti-CD3, anti-CD28, anti-IL-4, anti-IFN-γ antibodies, TGF-β, and IL-6 for 3 days. The cells were re-stimulated with phorbol 12-myristate 13-acetate (PMA) and ionomycin in the presence of GolgiStop for 4 h to detect intracellular IL-17. The percentage of IL-17^+^ cells among CD4^+^ cells was analyzed using flow cytometry. Values are shown as percentages of positive cells (**C**). Levels of IL-17 in the culture supernatant were determined using ELISA (**D**). **E** Splenic CD4+ T cells (5 × 10^6^ cells/5 ml) from C57BL/6 mice were untreated (Nil) or treated with IL-6 for 30 min or anti-CD3/anti-CD28 Ab for 12h in the presence of EC-18 and the levels of *p*-STAT3 (Tyr705 or Ser727) were measured by immunoblotting, respectively. Data are representative of three independent experiments. **F–H** At 56 days after the first immunization with CII, splenic CD4^+^ T cells (5 × 10^5^ cells/500 μl) were stimulated with EC-18 (10 or 100 μg/ml) or vehicle for 2 h and then cultured under Th17-activating conditions (**F**), with anti-CD3 and anti-CD28 antibodies (**G**), or under regulatory T cell (Treg)-polarizing conditions with anti-CD3, anti-CD28, anti-IL-4, anti-IFN-γ antibodies, and TGF-β (**H**) for 3 days. Levels of IL-17 (**F**) and IL-10 (**G, H**) in the culture supernatant were determined using ELISA. Data are presented as the means ± SEM of three independent experiments. *P < 0.05, **P < 0.01, ***P < 0.001 *vs*. vehicle-treated control

To determine the effects of EC-18 on the regulation of immune cells in vitro, murine splenocytes from naïve mice were cultured with EC-18 in the presence of LPS for 4 days. Treatment with EC-18 reduced the production of IL-6 and IL-1β in the culture supernatants of splenocytes (Fig. 5A). Furthermore, treatment with EC-18 reduced the secretion of IgG in the culture supernatants of splenocytes (Fig. 5B). To determine whether EC-18 could control the secretion of IL-17 in T cells, isolated splenic CD4+ T cells from naïve mice were cultured with or without EC-18 under Th17-polarizing condition. EC-18-treated CD4+ T cells were less prone to differentiate into Th17 cells compared to untreated cells, although there was no statistical significance (Fig. 5C). However, treatment of EC-18 dose dependently suppressed the amount of IL-17 in the culture supernatant of CD4+ T cells compared to untreated cells (Fig. 5D). To determine whether EC-18 can directly act on STAT3 phosphorylation, mouse splenic CD4+ T cells from naïve mice were stimulated with IL-6 or anti-CD3/anti-CD28 Ab in the presence of EC-18 and the levels of *p*-STAT3 (Tyr705 or Ser727) were measured, respectively. As shown in Fig. 5E, treatment with EC-18 inhibited IL-6-stimulated phosphorylation of STAT3 (Y705) in splenic CD4+ T cells. EC-18 also inhibited STAT3 phosphorylation (S727) in murine splenic T cells through anti-CD3/anti-CD28 Ab stimulation (Fig. 5E). Next, we used splenic CD4+ T cells from CIA mice to determine whether EC-18 treatment had a regulatory effect on T cells of CIA mice. Treatment with EC-18 reduced the secretion of IL-17 in the supernatant of splenic CD4^+^ cells from CIA mice under Th17-polarizing condition (Fig. 5F). The secretion of the anti-inflammatory cytokine IL-10 in the supernatant of splenic CD4^+^ cells from CIA mice was increased by EC-18 under anti-CD3 and anti-CD28 Ab stimulation (Fig. 5) Gand Treg-polarizing conditions (Fig. 5H).

### EC-18 decreases the production of inflammatory cytokines in human PBMCs by downregulating STAT3 phosphorylation

**Fig. 6:**
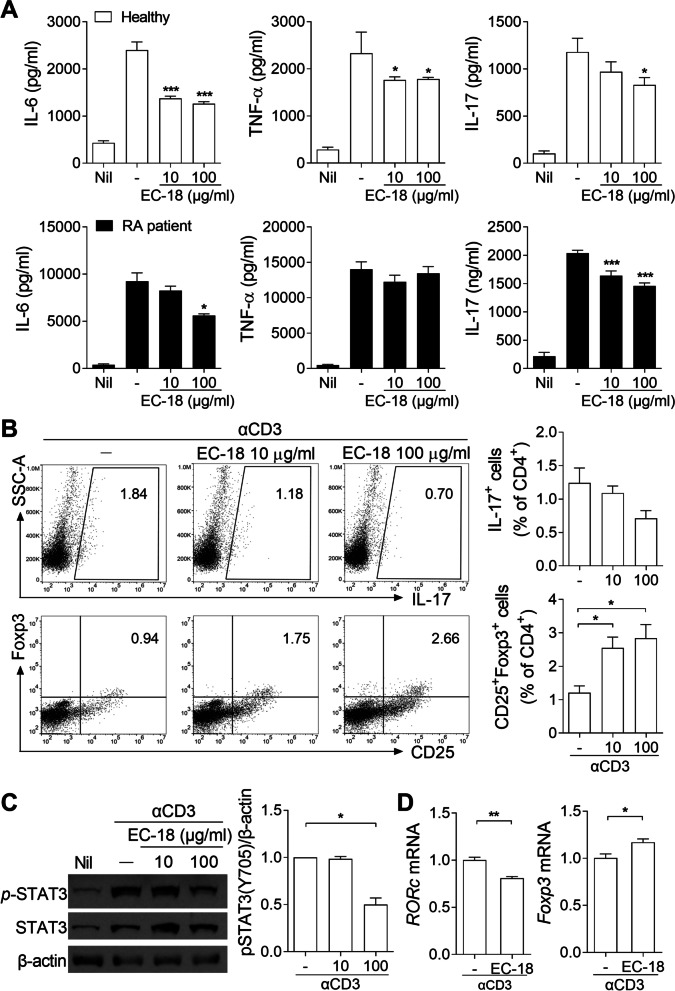
EC-18 suppressed the production of inflammatory cytokines in human cells by reducing signal transducer and activator of transcription (STAT) 3 phosphorylation. **A** Peripheral blood mononuclear cells (PBMCs) (1 × 10^6^ cells/1 ml) from healthy controls (*n* = 3, white bar) or patients with RA (*n* = 4, black bar) were cultured with EC-18 (10 or 100 μg/ml) or vehicle for 2 h and then untreated (Nil) or treated with anti-CD3 Ab for 3 days. Levels of IL-6, TNF-α, and IL-17 in the culture supernatant were determined using ELISA. **B** PBMCs (1 × 10^6^ cells/1 ml) from healthy controls were cultured with EC-18 (10 or 100 μg/ml) or vehicle for 2 h and then untreated (Nil) or treated with anti-CD3 Ab for 3 days. To detect intracellular IL-17, the cells were re-stimulated with PMA and ionomycin in the presence of GolgiStop for 4 h. The percentages of IL-17^+^ or CD25^+^Foxp3^+^ cells among CD4^+^ cells were analyzed using flow cytometry. Values are percentages of positive cells. **C** PBMCs (5 × 10^6^ cells/5 ml) from healthy controls were cultured with EC-18 (10 or 100 μg/ml) or vehicle for 2 h and then untreated (Nil) or treated with anti-CD3 Ab for 12 h. *p*-STAT3 (Tyr705) and STAT3 levels were determined via western blot analysis. Data are representative of three independent experiments. **D** PBMCs (1 × 10^6^ cells/1 ml) from healthy controls were cultured with EC-18 (1 μg/ml) or vehicle for 2 h and then with or without anti-CD3 Ab for 48 h. RORc and Foxp3 mRNA expression was determined using real-time polymerase chain reaction (PCR). Data are presented as the means ± SEM of three independent experiments. *P < 0.05, **P < 0.01, ***P < 0.001 *vs*. vehicle-treated control

To determine whether EC-18 could reduce the production of inflammatory cytokines in human cells, PBMCs from healthy controls and patients with RA were cultured with EC-18 in the presence of anti-CD3 Ab for 3 days. The amounts of IL-6, TNF-α, and IL-17 in the culture supernatant of PBMCs from healthy controls were significantly reduced by EC-18 (Fig. 6A). The levels of secretion of these inflammatory cytokines upon stimulation with anti-CD3 Ab were higher in the PBMCs of patients with RA compared to those from healthy subjects, and EC-18 effectively inhibited the production of IL-6 and IL-17 (Fig. 6A). To investigate whether EC-18 could modulate the balance between Th17 cells and Tregs, PBMCs from healthy controls were cultured with EC-18 and stimulated with anti-CD3 Ab for 3 days. EC-18 treatment tended to decrease the frequency of Th17 cells while increasing the frequency of Tregs in a dose-dependent manner (Fig. 6B). To evaluate the effects of EC-18 on the regulation of STAT3 phosphorylation, PBMCs from healthy controls were cultured with EC-18 and stimulated with anti-CD3 Ab for 12 h. Treatment with EC-18 reduced the level of *p*-STAT3 (Tyr705) compared to that in vehicle-treated controls (Fig. 6C). EC-18 reduced the mRNA expression of RORc, a transcriptional regulator of Th17 cells, but increased the mRNA expression of Foxp3, a transcriptional regulator of Tregs (Fig. 6D).

### EC-18 inhibits osteoclastogenesis in mouse and human cells

**Fig. 7:**
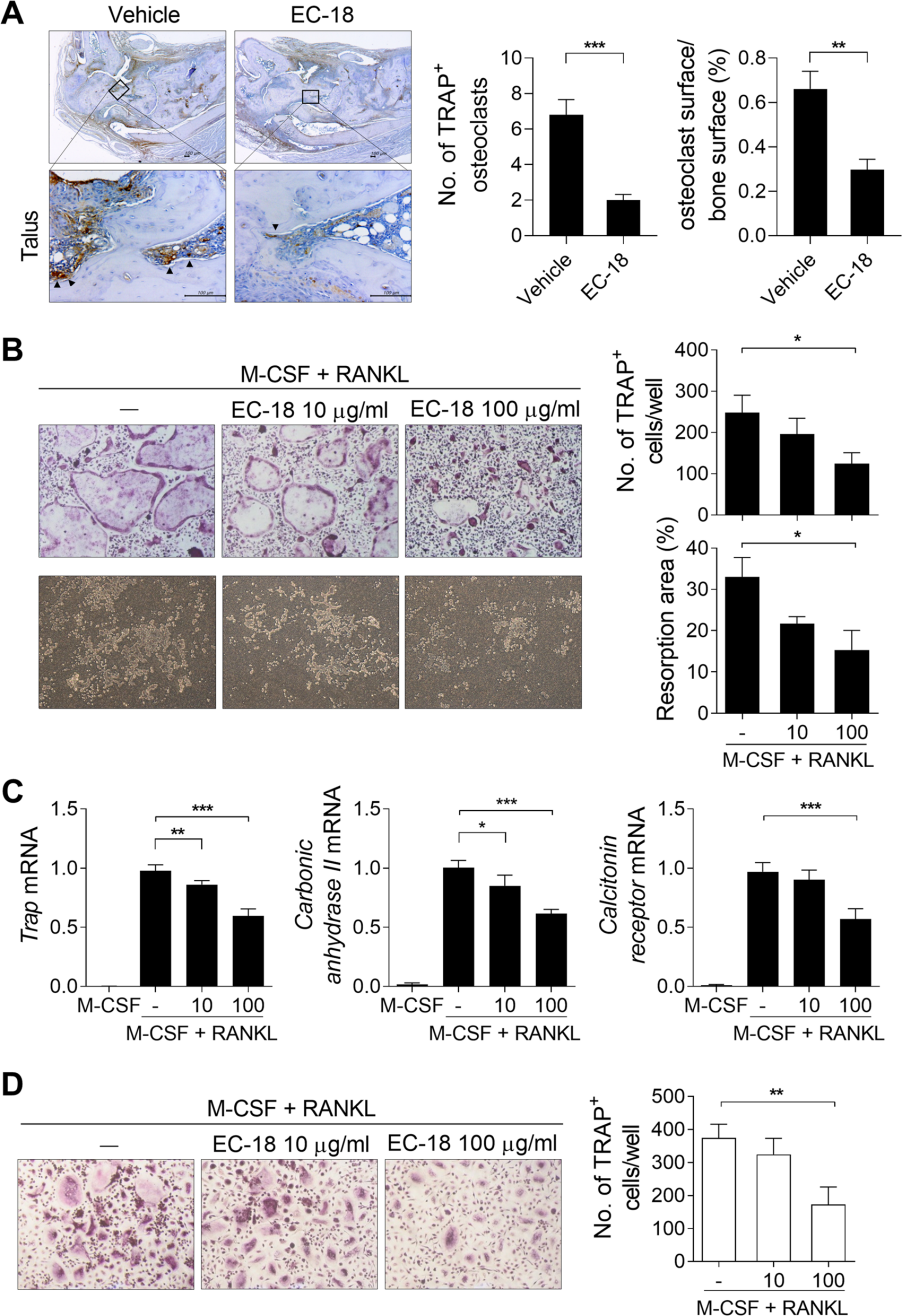
EC-18 inhibited osteoclastogenesis in mouse and human cells. **A** The vehicle or EC-18 (125 mg/kg) was administered to mice once daily from day 21 after the first immunization. At 70 days after the first immunization with CII, sections of joint tissues were stained with antibodies against TRAP (original magnification ×40 or ×400) (*n* = 5/group). Representative histological features are shown. The graphs present the number of TRAP-positive osteoclasts (left) and quantitative analysis of osteoclast surface/bone surface (right) in the talus endosteal surface of joint section. **B, C** Bone marrow-derived monocytes/macrophages (BMMs) (1 × 10^5^ cells/500 μl) from normal C57BL/6 mice were cultured with macrophage colony-stimulating factor (M-CSF) for 3 days. Then nonadherent cells were washed out and adherent cells were cultured in the presence of M-CSF and receptor activator of nuclear factor kappa-Β ligand (RANKL) with EC-18 (10 or 100 μg/ml) or vehicle for 4 days. Cells were stained to assess tartrate-resistant acid phosphatase (TRAP) activity, and the number of multinucleated TRAP^+^ cells was determined (upper). Osteoclasts were generated in OAAS plates in the presence of EC-18 for 6 days to assess their functional activity (lower, original magnification, ×100). Resorbed areas were examined using the Tomoro analySIS TS Lite program (**B**). The mRNA levels of TRAP, carbonic anhydrase II, and calcitonin receptor were determined using real-time PCR (**C**). **D** Human PBMCs from healthy controls (5 × 10^5^ cells/500 μl) were incubated for 2 h and then the adherent cells were cultured with M-CSF for 3 days. Then the cells were stimulated with EC-18 (10 or 100 μg/ml) or vehicle in the presence of M-CSF and RANKL for 9 days. Cells were stained to assess TRAP activity, and the number of multinucleated TRAP^+^ cells was determined. Data are presented as the means ± SEM of three independent experiments. *P < 0.05, **P < 0.01, ***P < 0.001 *vs*. vehicle-treated control

To determine the effects of EC-18 in osteoclastogenesis, tissue sections from EC-18- or vehicle-treated CIA (Fig. 3) were stained for TRAP. As shown in Fig. 7A, the number of TRAP^+^ osteoclasts and percentage of osteoclast surface/bone surface decreased in the talus endosteal surface, where bone is resorbed by osteoclasts [33], of the joint tissue from EC-18-treated CIA compared to vehicle-treated CIA mice. Next, we investigated the effects of EC-18 on in vitro osteoclast formation. BMMs isolated from normal C57BL/6 mice were cultured with M-CSF and RANKL in the presence or absence of EC-18. Treatment of murine BMMs with EC-18 effectively reduced their differentiation into TRAP^+^ mature osteoclasts as well as resorbed area compared with untreated control cells (Fig. 7B). Treatment with EC-18 also inhibited the expression of osteoclastogenic markers, such as TRAP, carbonic anhydrase II, and calcitonin receptor (Fig. 7C). To examine whether EC-18 could modulate the differentiation of osteoclasts in human cells, PBMC-derived monocytes from healthy controls were cultured with M-CSF and RANKL in the presence or absence of EC-18. Treatment with EC-18 inhibited osteoclast formation compared with the untreated controls (Fig. 7D).

## Discussion

In this study, we investigated the preventive effects on RA of EC-18, which is chemically synthesized from glycerol, palmitic acid, and linoleic acid, but is chemically identical to naturally derived EC-18. Our results showed that the application of EC-18 in vivo significantly prevented the clinical and histological severity of experimental arthritis, similar to tofacitinib and baricitinib, in CIA. Moreover, combined treatment with EC-18 plus MTX exhibited synergistic effects for the improvement of CIA compared to treatment with each of these drugs alone. Mechanistically, EC-18 effectively suppressed in vitro production of inflammatory cytokines, including IL-6, IL-1β, IL-17, and TNF-α, in immune cells and inhibited the osteoclast differentiation of mouse and human cells.

In traditional oriental medicine, Sika deer antler is considered to have beneficial therapeutic properties for the treatment of a wide variety of medical conditions, including mastitis, uterine fibroids, and mammary hyperplasia [19]. Sika deer antler contains a number of ingredients, among which it has recently been shown that monoacetyldiaglyceride has pharmacological properties, including hematopoietic and antitumor effects [21, 34, 35]. Although EC-18 was initially approved as a functional food in Korea, the United States FDA approved EC-18 for the treatment of severe chemotherapy-induced neutropenia in patients with advanced breast cancer being treated with intermediate febrile neutropenia risk chemotherapy [36]. In this study, we evaluated the efficacy of experimental arthritis treatment using chemically synthesized EC-18 with an efficacy similar to that of naturally derived EC-18 [21, 22]. Consistent with previously reported results [37], in this study we demonstrated that treatment with EC-18 ameliorated the development of experimental arthritic mice, and this effect was similar to that of treatment with tofacitinib or baricitinib, which are orally administered JAK inhibitors with low molecular weights.

MTX is a first-line DMARD that acts by disrupting the folic metabolic pathway and is a competitive inhibitor of the folate-dependent enzyme dihydrofolate reductase [31, 32, 38]. MTX has been shown to alleviate articular damage in RA and play a role in humoral and cellular immune responses [39, 40]. However, the clinical use of MTX has several side effects, including nausea, vomiting, anemia, neutropenia, pulmonary fibrosis, diarrhea, dermatitis, bone marrow depression, mucositis, bruising, and hepatitis [31, 32, 41]. Neurath MF et al. reported that MTX prevented CIA by lowering TNF-α production from splenic T cells in vivo and in vitro [42]. In addition, the CIA improvement efficacy was maintained even when MTX was combined with apoptotic cell-based therapy, which controls chronic inflammatory disorders by lowering the proinflammatory state and inducing tolerance to auto-antigens [43]. We found that the combined treatment with EC-18 and MTX controlled the development of experimental arthritis than either drug alone. These results suggest that EC-18 has potential as an effective therapeutic strategy for MTX-insufficient responders. However, it was not observed that the combination of EC-18 and MTX significantly lowered autoreactive and arthritogenic IgG antibodies reactive to CII in the serum [44]. It is necessary to investigate the synergistic effect of EC-18 and MTX combined therapy by increasing the number of mice in the experimental group and varying the measurement time point.

In this study, we showed that EC-18 reduces the production of inflammatory cytokines including IL-17, IL-6, and TNF-α in vitro in murine splenocytes and in human PBMCs. In particular, EC-18 suppressed the level of *p*-STAT3. Kim et al. showed that EC-18 inhibits IL-6 production by blocking STAT3 activity through LPS stimulation in RAW 264.7 macrophages and fibroblast-like synoviocytes from RA patients [37]. These results suggest that EC-18 is effective in blocking the activity of STAT3, a component of the JAK downstream signaling pathway. In addition to inhibiting STAT3 activity through EC-18, we observed that EC-18 could also inhibit the level of *p*-NF-κB p65 in murine splenic nonCD4 cells (data not shown). Moreover, treatment with EC-18 lowered the number of cells expressing either IL-6 or TNF-α in the synovium of CIA mice. In the early stages of joint inflammation, activated NF-κB is observed in human synovial tissue, and NF-κB promotes constitutive IL-6 production in synovial fibroblasts from RA patients [45, 46]. Shin et al. reported that EC-18 suppressed the increases in neutrophils and the levels of inflammatory cytokines such as IL-6 and TNF-α in bronchoalveolar lavage fluid and attenuated IκB and NF-κB phosphorylation in lung tissues [23]. These evidence show that EC-18 may decrease IL-6 and TNF-α by controlling the activity of NF-κB. EC-18 also lowered the levels of IL-4, IL-5, and IL-13 in bronchoalveolar lavage fluid and expression of iNOS in lung tissue from OVA-challenged asthmatic mice [24]. Yoon et al. demonstrated that EC-18 inhibited the activity of STAT6, a transcriptional activator of IL-4 expression, in monocyte and T lymphocyte cell lines, and lowered the levels of IL-4 and IgE in mice with DNCB-induced atopy-like dermatitis [22]. As the study of how EC-18 acts on intracellular signaling pathways has not been well studied, further studies are needed on the molecular mechanism by which EC-18 acts within the pathogenic cells.

Our in vivo and in vitro results show that EC-18 may act on the osteoclast differentiation. There is a lot of evidence that the regulation of JAK/STAT is important for bone homeostasis, but it is not fully understood. Osteoclast-specific STAT3 deficiency caused decreased osteoclast differentiation and bone resorption, but increased bone mass in mice through physical association of STAT3 and c-Fos to regulate the transcription of NFATc1 [47]. Osteoblast-specific disruption of STAT3 caused an osteoporotic phenotype through decreased mineral apposition rate and bone formation rate [48]. Recently, Adam et al. demonstrated that JAK inhibition can repair bone erosion by enhancing osteoblast function and mineralization activity without affecting osteoclastogenesis [49]. These reports suggest that the action of STAT3 in bone homeostasis is very complex. In this study, we found that EC-18 could act directly or indirectly on the differentiation of osteoclasts involved in bone destruction in rheumatoid arthritis. Further studies on the action mechanisms of EC-18 in maintaining the balance between bone formation and bone resorption are needed. Our results were limited in that although a low-dose combination of MTX and EC-18 was found to have potential for preventing experimental arthritis, it is still necessary to study the mechanism by which combined treatment with EC-18 and MTX exerts a greater disease-improving effect compared to MTX treatment alone.

## Conclusion

Taken together, the data presented here showed that EC-18 alone or in combination with MTX was effective in preventing experimental autoimmune arthritis via the regulation of inflammatory cytokines and the frequency of Th17 cells in CIA and human patients. Furthermore, EC-18 was shown to modulate osteoclastogenesis in mouse and human cells. These findings suggest that EC-18 may have potential as a new therapy for RA. Future studies using models of T- and B cell-independent arthritis models such as collagen antibody-induced arthritis and serum transfer arthritis are needed for more specific clarification of the anti-inflammatory efficacy of EC-18.

## Data Availability

The datasets used and/or analyzed during the current study are available from the corresponding author on reasonable request.

## Abbreviations

RA: Rheumatoid arthritis
IL: Interleukin
TNF: Tumor necrosis factor
Th17: IL-17-producing T helper
STAT3: Signal transducer and activator of transcription 3
M-CSF: Macrophage colony-stimulating factor
NF-κB: Nuclear factor kappa-B
RANKL: Receptor activator of NF-κB ligand
JAK: Janus kinase
CIA: Collagen-induced arthritis
MTX: Methotrexate
CII: Type II collagen
TRAP: Tartrate-resistant acid phosphatase
H&E: Hematoxylin and eosin
TGF: Transforming growth factor
LPS: Lipopolysaccharide
PBMC: Peripheral blood mononuclear cell
BMM: Bone marrow-derived monocyte/macrophage
ELISA: Enzyme-linked immunosorbent assay
DMARD: Disease-modifying antirheumatic drug
